# Past history of stage I/II solid tumor malignancy impacts considerably on sepsis mortality: a propensity score matching analysis from the hellenic sepsis study group

**DOI:** 10.1186/s12879-019-4448-7

**Published:** 2019-10-07

**Authors:** George Dimοpoulos, Nikoletta Rovina, Maria Patrani, Eleni Antoniadou, Dimitrios Konstantonis, Konstantina Vryza, Glykeria Vlachogianni, Miltiades Kyprianou, Christina Routsi, Evangelos J. Giamarellos-Bourboulis, on behalf of the Hellenic Sepsis Study Group

**Affiliations:** 1 2nd Department of Critical Care Medicine, National and Kapodistrian University of Athens, Medical School, Athens, Greece; 2 Intensive Care Unit, 1st Department of Pulmonary Medicine, National and Kapodistrian University of Athens, Medical School, Athens, Greece; 3 Intensive Care Unit, Korgialeneion-Benakeion Athens General Hospital, Athens, Greece; 4 Intensive Care Unit, “G.Gennimatas” Thessaloniki General Hospital, Thessaloniki, Greece; 5 Intensive Care Unit, Theageneion General Hospital, Thessaloniki, Greece; 6 Intensive Care Unit, “Aghios Dimitrios” Thessaloniki General Hospital, Thessaloniki, Greece; 7 4th Department of Internal Medicine, National and Kapodistrian University of Athens, Medical School, Athens, Greece; 8 1st Department of Critical Care Medicine, National and Kapodistrian University of Athens, Medical School, Athens, Greece; 9 4th Department of Internal Medicine, ATTIKON University Hospital, 1 Rimini Street, 12462 Athens, Greece

**Keywords:** Cancer, ICU, Severity, Outcome

## Abstract

**Background:**

Whether past history of solid stage I/II inactive cancer has an impact on 28-day mortality of sepsis remains unclear. We aimed to determine the impact of history of stage I or II solid tumor malignancy in complete remission the last 3 years on sepsis outcome.

**Methods:**

Using the database of the Hellenic Sepsis Study Group from 1553 patients with sepsis admitted in the ICU, 83 patients with sepsis by Sepsis-3 definition with past-history of stage I/II inactive solid malignancy the last 3 years were depicted. A comparator group of 83 patients fully matched for age, severity, type of infection and comorbidities was selected by propensity score matching.

**Results:**

Mortality after 28 days was 37.3% in the comparator group and 54.2% in the solid tumor stage I/II group (odds ratio for death 1.98; p: 0.030). Following step-wise forward Cox regression analysis, septic shock (hazard ratio 1.80), acute renal injury (hazard ratio 2.06), history of coronary heart disease (hazard ratio 0.36) and history of stage I/II solid tumor malignancy (hazard ratio 1.79) were the only independent variables associated with 28-day mortality. Serum levels of procalcitonin and of soluble urokinase plasminogen activator receptor were similar between the two groups of comparisons.

**Conclusions:**

Past history of stage I/II solid malignancy is an independent risk factor for unfavorable outcome from sepsis the first 28 days.

## Background

Throughout the past decades the low survival rates of critically ill patients suffering from malignancies led clinicians to the decision to avoid admission of these patients in the Intensive Care Unit (ICU) [1]. The advances in early detection and management of hematological malignancies and solid tumors over the last years have resulted in an increased number of ICU admissions of cancer patients who had significantly lower mortality in comparison to the 1980’s and the 1990’s [2, 3]. Large multicenter studies have shown that these patients account for 15–20% of all ICU admissions [4, 5]. The main causes of ICU admission do not differ between patients with malignancies and the general population [6] with sepsis being the second most frequent reason for ICU admission of cancer patients following postoperative care [5].

It is largely conceived that cancer patients are more prone to sepsis and unfavorable outcome due to factors associated with their malignancies like chemotherapy-induced immunosuppression and metastasis-associated organ obstructions [7]. However, no data are available if history of cancer that remains inactive may per se become a comorbidity driving towards unfavorable outcome.

In order to reply to this question, we used the database of the Hellenic Sepsis Study Group (HSSG) (www.sepsis.gr) that includes a broad collection of clinical data from both non-ICU and ICU patients with sepsis. We aimed to analyze the impact of history of stage I or II solid tumor malignancies in complete remission the last 3 years on the 28-day mortality of patients with sepsis admitted in the ICU.

## Methods

### Patients

The HSSG is collecting since June 2006 clinical data of patients with clinically and/or microbiologically documented infections and systemic inflammatory response (SIRS) from 55 departments across Greece; 21 study sites are ICUs. This is done by a central protocol that has been approved by the Ethics Committees of the participating hospitals where the study sites are located. Adult patients are enrolled after written informed consent provided by the patients or first degree relatives in the case of patients unable to consent. Since March 2016 all patients in the database were re-classified into infection, sepsis and septic shock using the Sepsis-3 classification criteria [8]. Patients with febrile neutropenia and infection by the human immunodeficiency virus are excluded from the HSSG registry.

In January 2019, it was decided to investigate the role of history of inactive stage I/II lung and colorectal adenocarcinoma in the final outcome of sepsis among patients admitted for sepsis in the ICU. Investigators from all ICUs participating in the HSSG defined specific criteria for the selection of patients of the database for this analysis. These criteria were: a) admission in the ICU; b) sepsis defined by Sepsis-3 definition; c) history of stage I or II solid tumor in complete remission the last 3 years that did not predispose to infection; and d) lung cancer and colorectal cancer. Exclusion criteria were: a) any solid tumor malignancy of stages III or IV; b) any hematologic malignancy; c) any lymphoma; d) intake of chemotherapy; e) ICU admission due to postoperative care for cancer resection; and f) malignancy as direct predisposition to sepsis. The last exclusion criterion comprised, but was not limited, to the following conditions: acute ascending cholangitis due to biliary tract obstruction by tumor; acute ascending pyelonephritis due to ureter obstruction by tumor; lung infection due to bronchial obstruction by tumor; and intraabdominal infection developing after tumor resection.

Patients remaining after the application of the inclusion and exclusion criteria were divided into two groups; those with stage I/II solid tumor malignancy and comparators without any malignancy. Two fully matched groups were formatted with an equal number of patients using specific matching criteria. These criteria were age, SOFA score, APACHE II score, Charlson’s Comorbidity Index (CCI), presence of septic shock and type of infection. Matching was done by propensity score calculation.

Blood was sampled from all enrolled patients within the first 24 h from enrolment. Blood was collected into sterile and pyrogen-free and anticoagulant-free tubes (Vacutainer, Becton Dickinson, Cockeysville Md). Tubes were transported within 1 day by a courier service to the Laboratory of Immunology of Infectious Diseases of the 4th Department of Internal Medicine at ATTIKON University Hospital of Athens. Tubes were centrifuged and serum was kept refrigerated at -70 °C until assayed. Procalcitonin (PCT) was estimated in serum in duplicate by an immuno-time-resolved amplified cryptate technology assay (Kryptor PCT; BRAHMS GmbH, Henningdorf, Germany) with a functional assay sensitivity of 0.06 ng/ml. Concentrations of soluble urokinase plasminogen activator receptor (suPAR) were measured by an enzyme immunosorbent assay with a functional assay sensitivity of 2.1 ng/ml (suPARnostic, Virogates, Medicon Valley, Denmark).

The following information was recorded per patient: demographics, severity using the APACHE II score and SOFA score, comorbidities, microbiology, and administered antimicrobials. Patients were followed-up for 28 days. The selection and dose of administered antimicrobials for all patients was done according to the national guidelines that are published by the Hellenic Society for Infectious Diseases (www.loimoxeis.gr). The collection of data in each study site was confirmed by independent monitors.

### Study endpoints

The primary study endpoint was to determine whether history of stage I/II cancer is influencing 28-day mortality of patients with sepsis who are hospitalized in the ICU. The secondary endpoint was to define if there are differences in the concentrations of PCT and suPAR between the two groups.

### Statistical analysis

Comparisons between comparators and patients with history of stage I/II solid malignancy were done by the Fisher exact test for binary variables, by the Student’s t-test for parametric continuous variables and by the Mann-Whitney U test for non-parametric continuous variables. Survival was compared by the log-rank test. The odds ratio (OR) and 95% confidence intervals (CIs) were determined by the Mantel and Haenszel test. Variables differing significantly between survivors and non-survivors entered the equation of forward step-wise Cox regression analysis as dependent variables with 28-day mortality as the independent variables. Hazard ratios and 95% CIs were calculated. Concentrations of PCT and suPAR were expressed as median and 95% CIs. Any *p*-value below 0.05 was considered significant. Analysis was done using IBM SPSS statistics version 25.0.

## Results

**Fig. 1:**
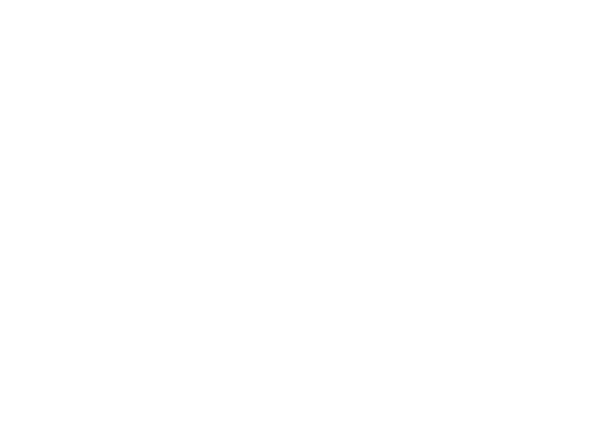
Study flow chart. Abbreviations ICU: intensive care unit; SIRS: systemic inflammatory response syndrome

The study flow chart is shown in Fig. 1. Using propensity score matching, two groups were formatted; comparators without malignancy (*n* = 83) and patients with history of stage I/II solid malignancy (*n* = 83). The two groups were fully matched for APACHE II score, SOFA score, CCI, age and organ dysfunctions. The time to the administration of the first dose of antimicrobials from the onset of sepsis or vasopressors did not differ between groups. Microbiological confirmation of the pathogen was available in 86 patients. Susceptibilities of the isolated pathogens to the administered antimicrobials did not differ between the two groups (Table 1).

**Table 1:** Characteristics of analyzed patients

	Sepsis without malignancy (*n* = 83)	Sepsis with stage I/II solid malignancy (*n* = 83)	*p*-value
Male gender (n, %)	56 (67.5)	53 (63.9)	0.744
Age (years, mean ± SD)	64.0 ± 18.4	69.10 ± 9.85	0.435
APACHE II score (mean ± SD)	21.2 ± 8.1	21.9 ± 6.9	0.782
SOFA score (mean ± SD)	8.47 ± 3.74	8.52 ± 3.39	0.915
CCI (mean ± SD)	4.70 ± 3.37	5.64 ± 2.27	0.057
White blood cells (/mm^3^, mean ± SD)	15,783.3 ± 8868.6	14,121.2 ± 8977.1	0.120
Infection site (n, %)			
Hospital-acquired pneumonia	26 (31.3)	25 (30.1)	1.00
Primary bacteremia	15 (18.1)	15 (18.1)	1.00
Intrabdominal infection	14 (16.9)	21 (25.3)	0.253
Community-acquired pneumonia	14 (16.9)	10 (12.0)	0.509
Acute pyelonephritis	5 (6.0)	3 (3.6)	0.720
Acute bacterial skin and soft tissue infection	4 (4.8)	0 (0)	0.120
Bacterial meningitis	1 (1.2)	1 (1.2)	1.00
Other	4 (4.8)	8 (9.6)	0.369
Organ dysfunction (n, %)			
Septic shock	56 (67.5)	56 (67.5)	1.00
Acute respiratory distress syndrome	44 (53.0)	46 (55.4)	0.876
Acute kidney injury	22 (26.5)	12 (14.5)	0.083
Disseminated intravascular coagulation	17 (20.5)	22 (26.5)	0.464
Bloodstream isolate (n, %)			
*Acinetobacter baumannii*	7 (8.4)	9 (10.8)	0.793
*Klebsiella pneumoniae*	3 (3.6)	5 (6.0)	0.720
*Pseudomonas aeruginosa*	3 (3.6)	5 (6.0)	0.720
*Staphylococcus aureus*	3 (3.6)	0 (0)	0.245
*Enterococcus faecalis*	3 (3.6)	3 (3.6)	1.00
*Enterococcus faecium*	1 (1.3)	3 (3.6)	0.621
Other	9 (10.8)	6 (7.2)	0.600
Urine isolate (n, %)			
*Klebsiella pneumoniae*	2 (2.4)	2 (2.4)	1.00
*Acinetobacter baumannii*	3 (3.6)	1 (1.3)	0.620
Tracheobronchial secretions isolate (quantity > 10^5^ cfu/ml)			
*Klebsiella pneumoniae*	4 (4.8)	8 (9.6)	0.370
*Pseudomonas aeruginosa*	6 (7.2)	7 (8.4)	1.00
*Acinetobacter baumannii*	14 (16.9)	22 (26.5)	0.187
*Staphylococcus aureus*	3 (3.6)	0 (0)	0.245
Other	6 (7.2)	4 (4.8)	0.746
Co-morbidities (n, %)			
Type 2 diabetes mellitus	22 (26.5)	16 (19.3)	0.356
Chronic heart failure	23 (27.7)	18 (21.7)	0.472
Chronic obstructive pulmonary disease	17 (20.5)	17 (20.5)	1.00
Chronic renal disease	11 (13.3)	6 (7.2)	0.306
Coronary heart disease	12 (14.5)	11 (3.3)	1.00
Vascular hypertension	18 (21.7)	21 (25.3)	0.715
Atrial fibrillation	9 (10.8)	8 (9.6)	1.00
Stroke	13 (15.7)	10 (12.0)	0.654
Time (hours) from sepsis onset to the administration of the first dose of antimicrobials (median, range)	5 (0–125)	6 (0–216)	0.112
Time (hours) from onset of vasopressors to the administration of the first dose of antimicrobials (median, range)	2.7 (0–55)	2.8 (0–168)	0.444
Susceptibility of the isolated pathogen to the administered antimicrobials based on antibiogram (n, %)	22 (26.5)	22 (26.5)	1.00

Abbreviations *APACHE* Acute physiology and chronic health evaluation, *CCI* Charlson’s comorbidity index, *SOFA* Sequential organ failure assessment

**Fig. 2:**
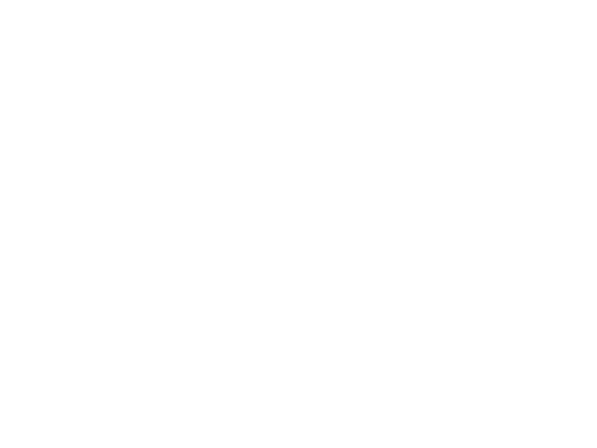
Survival of 83 patients with sepsis and history of stage I/II malignancy and of 83 fully-matched sepsis comparators. The log-rank test of comparison and the respective *p*-value are provided

Mortality after 28 days was 37.3% in the comparator group (31 out of 83 patients) and 54.2% in the solid tumor stage I/II group (45 out of 83) (Fig. 2). The OR for death among patients with history of solid tumor was 1.98 (95%CIs: 1.07–3.69; p: 0.030). Nil patients in the malignancy group died due to cancer.

At a next step, baseline demographics were compared between 28-day survivors and non-survivors. Variables being significantly different between the two groups of comparisons were septic shock, acute kidney and history of stage I/II solid malignancy that were more common among non-survivors; and history of coronary heart disease that was more common among survivors (Table 2). The impact of these four variables was confirmed after forward step-wise Cox regression analysis (Table 3). Analysis was repeated for these variables among the 86 patients with microbiologically confirmed infections. Susceptibility of the pathogen to the administered antimicrobials was included as another variable in the equation. History of stage I/II solid malignancy was again confirmed to be an independent variable associated with unfavorable 28-day outcome (Table 4).

**Table 2:** Differences in baseline characteristics between survivors and non-survivors

	Survivors (*n* = 90)	Non-survivors (*n* = 76)	*p*-value
Infection site (n, %)			
Hospital-acquired pneumonia	27 (30.0)	24 (31.6)	1.00
Primary bacteremia	13 (14.4)	17 (22.4)	0.226
Intrabdominal infection	14 (16.9)	21 (25.3)	0.253
Community-acquired pneumonia	12 (13.3)	12 (15.8)	0.665
Acute pyelonephritis	6 (6.7)	2 (2.6)	0.291
Acute bacterial skin and soft tissue infection	4 (4.4)	0 (0)	0.126
Bacterial meningitis	2 (2.2)	0 (0)	0.501
Other	3 (3.3)	0 (0)	0.251
Organ dysfunction (n, %)			
Septic shock	52 (57.8)	60 (78.9)	0.005
Acute respiratory distress syndrome	43 (47.8)	47 (61.8)	0.086
Acute kidney injury	12 (13.3)	22 (28.9)	0.020
Disseminated intravascular coagulation	17 (18.9)	22 (28.9)	0.144
Co-morbidities (n, %)			
Type 2 diabetes mellitus	21 (23.3)	17 (22.4)	1.00
Chronic heart failure	21 (23.3)	20 (26.3)	0.719
Chronic obstructive pulmonary disease	16 (17.8)	18 (23.7)	0.440
Chronic renal disease	9 (10.0)	8 (10.5)	1.00
Coronary heart disease	18 (20.0)	5 (6.6)	0.014
Vascular hypertension	23 (25.6)	16 (21.1)	0.583
Atrial fibrillation	11 (12.2)	6 (7.9)	0.445
Stroke	12 (13.3)	11 (14.5)	1.00
History of stage I/II solid malignancy	38 (42.2)	45 (59.2)	0.043

**Table 3:** Forward step-wise Cox regression analysis of variables associated with 28-day mortality

Variable	Hazard ratio	95% confidence intervals	*p*-value
Septic shock	1.80	1.01–3.22	0.046
Acute kidney injury	2.06	1.21–3.49	0.007
History of coronary heart disease	0.36	0.14–0.89	0.028
History of stage I/II solid malignancy	1.79	1.13–2.85	0.014

Variables significantly different between survivors and non-survivors of Table 2 entered the equation as dependent variables. Variables remaining significant after four steps of analysis are shown

**Table 4:** Cox regression analysis of variables associated with 28-day mortality among patients with microbiologically confirmed infections

Variable	Hazard ratio	95% confidence intervals	*p*-value
Septic shock	1.45	0.67–3.15	0.345
Acute kidney injury	2.06	0.94–4.55	0.073
History of coronary heart disease	0.74	0.25–2.19	0.587
Susceptibility of the pathogen to the administered antimicrobials	0.54	0.26–1.11	0.096
History of stage I/II solid malignancy	2.72	1.37–5.40	0.004

Variables described in Table 3 entered the equation along with susceptibility of the isolated pathogen to the administered antimicrobials

**Fig. 3:**
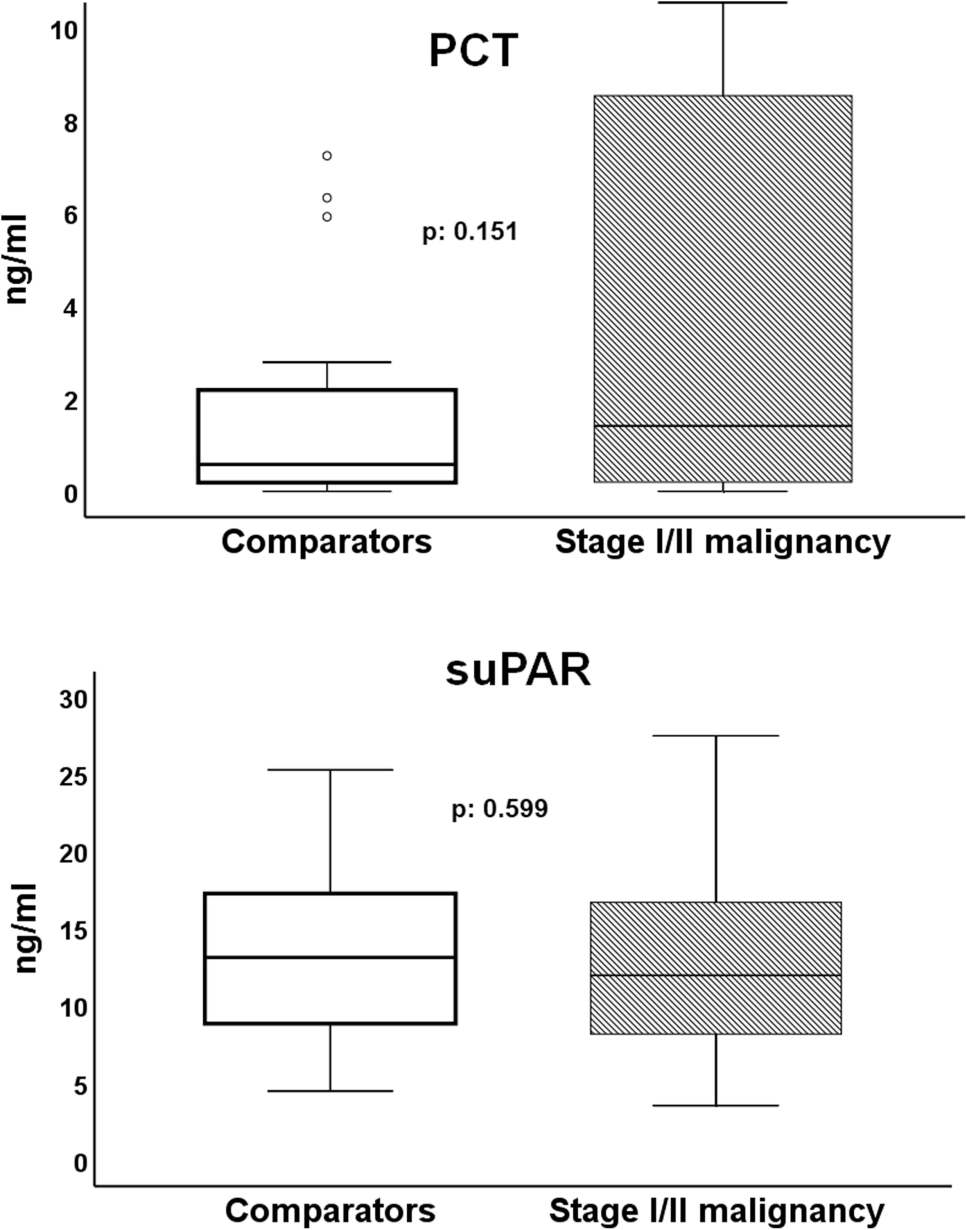
Concentrations of procalcitonin (PCT) and of soluble urokinase plasminogen activator receptor (suPAR) of 83 patients with sepsis and history of stage I/II malignancy and of 83 well-matched sepsis comparators. The respective *p*-values of comparisons are provided

No differences in PCT and suPAR concentrations were found between the two groups of comparison (Fig. 3).

## Discussion

The present study used a unique study design and tried to provide answers to a common medical question that remains unanswered. The medical question is whether past-history of stage I/II solid tumor malignancy that remains inactive impacts on 28-day mortality. Findings were affirmative but in order to answer this question an extensive filtering process was needed. During this process, only patients without treatment for their malignancy and without any apparent role of the malignancy as a factor predisposing to sepsis were analyzed. They were compared to patients matched not only for age but also for severity, organ dysfunction, type of infection and comorbidities. A similar approach has never been done before.

Available studies that investigate the outcome of patients with cancer and sepsis enroll only patients suffering from cancers and they do not narrow their analysis on stage I/II patients. Instead they enroll all patients irrespective of the presence or not of metastasis, of the intake or not of chemotherapy and of the presence of neutropenia. Analysis ends-up that 28-day mortality is dependent on the severity of organ failures where neutropenia post-chemotherapy is another independent prognostic variable for unfavorable outcome [5, 9–12]. This is in line with the results of our Cox regression analysis showing organ dysfunction as a salient risk factor for unfavorable outcome. However, most of patients with malignancy bear the risk for infections by multidrug-resistant bacteria probably due to frequent hospitalizations leading to colonization by that species [13]. It is evident that this has a major impact on 28-day outcome through the intake of inappropriate antimicrobial therapy [13, 14].

Two major limitations of the study need to be addressed; matching for appropriateness of antimicrobial therapy and quality of care between study sites. Regarding administered antimicrobials therapy it needs to be outscored that although not all variables were addressed, patients with history of stage I/II solid tumor malignancy and comparators did not differ in timing of administration and susceptibilities of the isolated pathogens to the administered antimicrobials.

There are two more studies that are trying to address the same question but that have not succeeded to provide comparators with similar severity and comorbidities. The first study is coming from Lebanon and it is retrospective analysis of 176 oncological patients with active hematologic or solid tumor malignancy on chemotherapy or radiation therapy. These were compared to another 176 non-oncological patients coming from the same database. Although the hospital mortality of the oncological patients was significantly greater than the non-oncological patients, their severity as defined by their vital signs and requirement for vasopressors was also significantly greater than the non-oncological patients [15]. The second study is a retrospective analysis of the REGARDS database. Cancer survivors were compared to patients without cancer history with regards to hospital death and to the time until the first sepsis event. Cancer survivors were defined as patients without need of chemotherapy or radiation the past 2 years. Results showed that the time to the first sepsis event was significantly shorter among cancer survivors and that in the case of infection hospital mortality was 8.29% among cancer survivors and 3.93% among non-cancer comparators. However, the two groups were not adequately matched since cancer survivors were older and they were presenting with significantly more comorbidities than the comparators. Moreover, the analysis did not exclude patients with stage III/IV malignancies [16].

The mechanism explaining why a past history of stage I/II solid tumor malignancy predisposes to death in case of sepsis cannot be derived by our findings. It may be argued that cancer survivors are more immune debilitated due to either cancer itself or to previous cancer treatment. This is compatible with the difference of the survival curves between cases and comparators of our study starting to separate by day 7. It is remarkable that measured PCT and suPAR are similar with comparators showing that in these patients biomarkers need to be interpreted as in the non-cancer populations. It needs to be underscored that suPAR is a non-specific biomarker of mortality irrespective of causality [17]. Although suPAR is early increased among cancer patients with unfavorable outcome [18], results presented here indicate that past history of malignancy cannot affect circulating levels more than the septic process.

Another interesting finding of the present study, although bizarre at first glance, is the protective role of coronary heart disease. It is not the first time where a similar finding is reported. An analysis of the National Inpatient Sample database on 7.1 million of hospitalizations for severe sepsis during the period 2007 to 2013 in the United States reported 0.15% incidence of cases of Tako-Scubo cardiomyopathy (TCC). TCC is characterized by hypokinesia, akinesia or dyskinesia of the left ventricle in the absence of coronary obstruction. TCC was an independent protective factor from 28-day mortality [19]. The low number of the studied patients, probably influencing the power of the results, signifies that this finding should be interpreted with caution.

## Conclusions

The present study revealed that past history of stage I/II solid malignancy is an independent risk factor for 28-day mortality in sepsis. Results suggest that past history of stage I/II malignancy should be a variable taken into consideration in the endpoint analysis of sepsis trials.

## Data Availability

Data are available upon request. Please contact the corresponding author E. J. Giamarellos-Bourboulis.

## Abbreviations

APACHE: Acute physiology and chronic health evaluation
CCI: Charlson’s comorbidity index
CI: Confidence interval
HSSG: Hellenic Sepsis Study group
ICU: Intensive care unit
OR: Odds ratio
PCT: Procalcitonin
SOFA: Sequential organ failure assessment
suPAR: Soluble urokinase plasminogen activator receptor
TCC: Tako-Scubo cardiomyopathy
